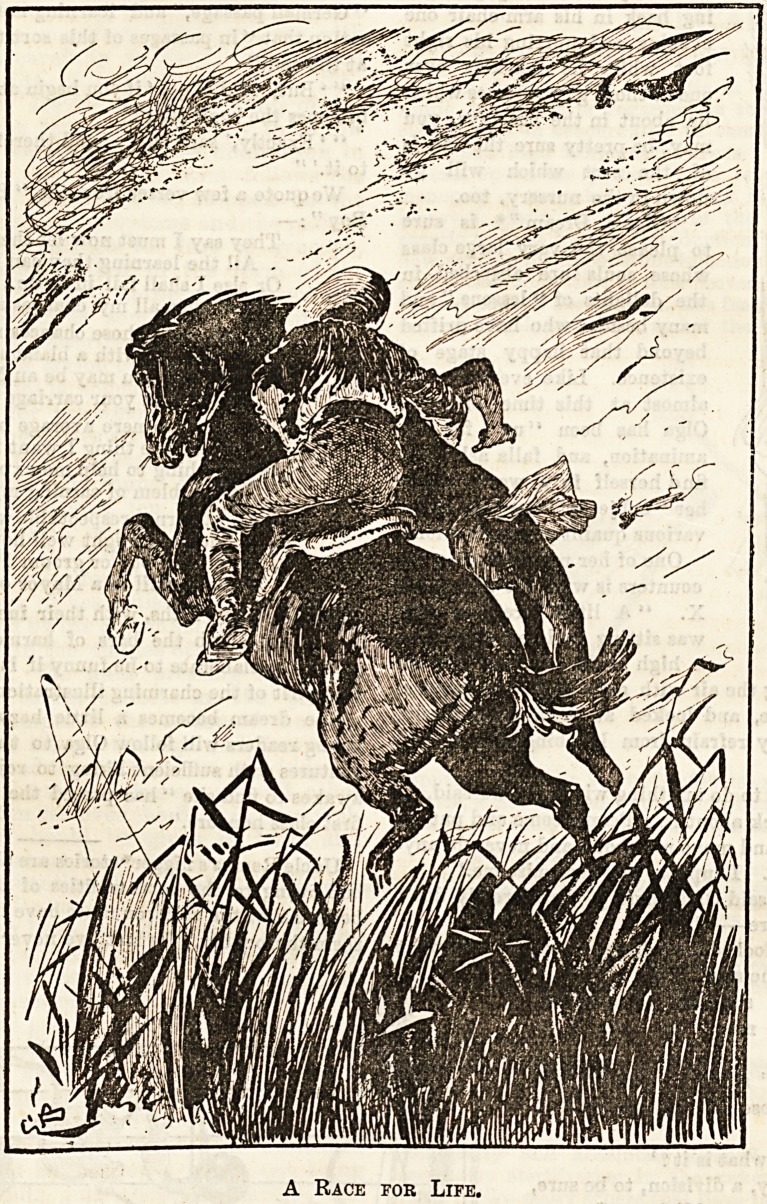# Christmas Fare for the Children

**Published:** 1892-12-31

**Authors:** 


### Christmas Fare for the Children.

Most people who are worth anything at all can turn into children again on occasions, and there is no better test of a child's book or a toy than its power to amuse the "grown-ups." Have you never seen uncles, cousins, and aunt-screaming with delight over baby's pile of presents, spinning tops, blowing trumpets, winding-up mice, and so forth, while baby himself sits sucking his thumb in lordly indifference? And if you detect papa leaning back in his arm-chair one evening and nursing his right foot, while he chuckles over one of those gay volumes which lie about in the holidays, you may be pretty sure the fun is of the sort which will go down in the nursery, too.

"Olga's Dream"* is sure to please the very large class whose souls are absorbed in the delights of "lessons," and many besides who have drifted beyond that happy stage of existence. Like everyone else almost at this time of year. Olga has been "up" for examination, and falls asleep to find herself in a world where her "subjects" reappear under various quaint personifications.

One of her most amusing encounters is with the ill-treated X. "A little creature who was sitting huddled up against a high wall, rocking himself to and fro, and filling the air with sobs and lamentations. He rose as they spoke, and looked at them suspiciously, and Olga could hardly refrain from laughing at his odd appearance.

"'If you have come to do anything with me,' he said, 'I wish you would be quick about it. People come and put me down and take me up and move me about, and never do anything with me after all. I suppose you recognise me.'

"'I am very sorry,' said Olga timidly, 'but I really don't think I have the pleasure——'

"The little creature looked very disappointed. 'I thought you would be sure to,' he said, 'I am X.'

"'Oh,' cried Olga, suddenly enlightened, 'I do believe I know now. You are X in Algebra.'

"'Why, of course,' said X, 'where else should I be? I suppose you see the wall in front of you?'

"'Yes,' said Olga, 'what is it?'

"'What is it? Why, a division, to be sure, and that higher piece is an addition. Can you see over it?"

"'I think so,' said Olga, standing on tiptoe. 'Oh yes, I see a number of little shelves with statues in them.'

"'They are generally called brackets and figures,' said X.

"'And beyond that I can see part of a tree,' said Olga.

"'Ah!' said X, 'so you can just see the higher branches, can you?' He sat down, and began to rock himself to and fro. 'Perhaps you understand now,' he said, 'why I am so unhappy. It is a dreadful life that I have of it here, I can assure you. There is never any peace for me. They put me first on one side and then on another; they badger and tease me, and kick me about, and use me just to suit their convenience, and then at the end, after I have done all the hard work, they cast me aside altogether, and let a figure take my place and have all the honour and glory. A nasty, common figure.'"

There is a pretty illustration of Olga struggling through a "German passage," and learning not without some mystification that "in passages of this sort the best plan is to begin at the end."

"'But,' said Olga, 'if you begin at the end, surely the end becomes the beginning?'

"'Exactly,' said Dick, 'and therefore there seems no end to it.'"

We quote a few verses from the "Ballad of the Pale-faced Boy":—

> They say I must now let them cram
> All the learning they can in my head,
> Or else I shall fail in exam.,
> And then all my chances are fled.
>
> If I ask what those chances may be,
> They reply with a bland sort of smile,
> "Some day you may be an M.P.,
> And ride in your carriage in style."
>
> But mine's a mere average brain,
> And the one thing I want in my head,
> Is something to help me explain
> The problem of earning my bread.
>
> I might earn a respectable wage,
> A success I might well hope to be
> If as plough-boy or groom I engage.
> I should fail as a Mayor or M.P.

The consecutive fifths, with their inevitable jar, attempting in vain to climb the bars of harmony, is a bit of fooling rather too elaborate to be funny if it were not redeemed by the spirit of the charming illustration which we reproduce.

The dream becomes a little hazy at the end, but most young readers will follow Olga to the conclusion of her adventures with sufficient liking to rejoice with her when she awakes to find she "has passed the examination and taken first class honours."

---

Uncle Remus's nigger* stories are always excellent reading, but owing to the eccentricities of the orthography we can believe that the children must have them read aloud to enjoy them thoroughly. In fact, we never open one of Mr. J. C.

Harris' folk-lore stories without wishing we could hav changed places with "the little boy," and heard the expressive negro dialect with its wealth of humour and pathos from the lips of Uncle Remus himself. This is specially the

*"Olga's Dream," by Morley Chester. Skeffington and Son. Illustrated by Harry Furniss and Irving Montagu.

*"Daddy Jake the Runaway, and Short Stories Told After Dark by Uncle Remus" (J. C. Harris). T. Fisher Unwin.

case in his illustrations of bird language, which good as they are in print rouse a hungry longing to hunt up Uncle Remus and make him "do it" for us as he did for the child, giving his voice a far away but portentous sound, the intonation being a weirdly exact intonation of the hooting of a large swamp owl. The italicised words give a faint idea of this intonation.

One such passage we cannot resist transcribing, containing the midnight adventures of a gentleman known to Uncle Remus as Becky's Bill. We should warn our readers that the difficulty of the dialect lies more in appearance than in reality—

"Wen dat nigger wuz growin' up he went frolickin' round, en one night he come froo de Two Mile Swamp. He come 'long he did, and the fust news you know a great big ole owl flew'd up in a tree en snap he bill des like somebody crackin' a whip. Becky's Bill make like he ain't take no notice, but he sorter men' he gait. Presently ole Mr. Owl flewed up in er tree little ways ahead en smack he mouf. Den he holler out—

"'*Who* cooks—*who* cooks—*who* cooks fer *you*—all?'

"Becky's Bill move on—he make like he ain't year nothing. Ole Mr. Owl holler out—

"'*Who* cooks—*who* cooks—*who* cooks fer *you*—all?'

"By dat time Becky's Bill done get sorter skeered, en he stop en say—

"'Well, sir, endurin' er de week, mammy, she cooks, but on Sundays, and mo' speshually ef dey got comp'ny, den ole Aunt Dicey, she cooks.'

"Ole Mr. Owl he ruffles up he fedders, he did, en smack he mouf, en look down at Becky's Bill, en 'low: '*Who* cooks—*who* cooks—*who* cooks fer *you*—all?' Becky's Bill he take off he hat, he did, and 'low, sez ee: 'Well, sir, its des like I tell you. Mos' ingenerlly endurin' er de week, mammy, she cooks, but on Sundays, mo' speshually wen they got comp'ny, ole Aunt Dicey she cooks.'

"Ole Mr. Owl he keep axin, en Becky's Bill kept on tellin', twel bimeby, Becky's Bill, he got skeered, en tired, en mad, en den he lept out fum dar and en he run home like aquarter hoss."

A Race for Life.

Among books for boys we welcome a cheap edition* of "In Quest of Gold" as an excellent story of adventure. Bushranging, gold digging, wild rides, and desperate encounters with the natives make up an attractive volume, and the illustrations are as spirited as the text. The race with the bush fire of the two Australian lads and their native servant is told with the dash and vigour of a horseman describing from the life.

"Knowing full well the horrors of a great bush fire the boys put their horses to their best speed and galloped on. The quivering limbs of the beasts, their dilated nostrils, and wildly starting eyes showed how greatly they feared the dreaded element. Now it was that they began to pass numbers of animals all hurrying and rushing along in abject terror in the opposite direction to the horsemen. Kangaroos and wallabies progressing by great leaps; emus flapping their inefficient wings to help them in their flight; bush rats and smaller creatures scuttling along by the side of wriggling snakes and currish dingoes. Overhead were flocks of parrots, pigeons, cockatoos, and other bush birds, all flying away from the great cloud of rolling smoke and flame that seemed to stride with enormous steps after the flying creatures. Every moment as the three hurried on the heat became greater. They could now plainly hear the wild roar and crackling of the awful fire as it consumed everything before it in its devastating march, and the burning air that came in puffs and beat upon them scorched and withered them. Their very eyeballs seemed to dry within their sockets and the smarting lids when they closed them, hardly kept out the awful glare. The natural light of day was gone, for the whole sky was covered with one vast cloud of lurid smoke, and everything looked red and burning from the ruddy light of the sweeping flames." The excitement is well sustained to the end, where the last horseman, lifting the native in his arms, dares his horse at the line of fire which separates him from the safety of the barren rock they

* "In Quest of Gold." By Alfred St. Johnston. (Cassell and Co.)

are aiming at, and lands on the glowing ashes of the ground the fire had just passed over.

* Messrs. Dean and Son have brought out some new picture-books with nursery rhymes for quite the tinies, which wil cause endless delight as long as the little fingers can be kept from the work of destruction. By a contrivance of fine string attached to the various parts of the picture each full-page illustration starts into relief as soon as the page is turned, and train, windmill, or ship appear for the moment in motion.

† The "Nursery Rhymes" issued by the same house in exact imitation of Fry's chocolate, may possibly be the cause of some disappointment as "only a book" when dug out of the Christmas stocking by eager hands in search of "something good."

‡ An exact reprint of "Old Mother Hubberd," as issued in 1793, with modern illustrations in contrast with the old ones, is pretty and out of the common. We fear no one will hesitate which century to prefer.

In the hunt for Christmas presents we noted several new things at the toy exhibition at the Royal Aquarium. The fact is, unhappily, only too evident that a great branch of industry is diverted from English hands by the greater skill and ingenuity of the German and American artificer. The commoner sorts of dolls and toys, certain heavy carts and horses, and a good deal of indifferent bone-work represent the English part of the show. Some of these are shown in process of manufacture. We should, perhaps, add to these the "Strongest Men in the World," a popular Jack-a-Dandy of Lord Salisbury and Mr. Gladstone boxing. But the English work is common. It has all the evils which attend incessant and mechanical reproduction of one model, allowing no scope whatever for individuality. Would it be too much, in view of the enormous sale of toys of every description (but chiefly of foreign make), to require instruction in toy-making to take a regular place in the system of technical education? Invention might be stimulated by prizes, and many whose trade is precarious would find the art of using their fingers once acquired a useful one to fall back upon in hard times. The Germans, who make toys from early childhood, excel in beauty of workmanship. The Emperor William's Hunting Château is a beautifully-designed model, with stables, containing horses and groom below, and a daintily-fitted bed-room and study above. The Castle of King Ludgwig of Bavaria is also well executed, and a gay mechanical scene from Lake Como, warranted to go on working for two hours, would be a boon in many a nursery, might we not say in many a children's ward, where the afternoon hours drag on slowly and it requires all "sister's" persevering brightness to restrain the fretful whine of the little tired sufferers.

But for thorough ingenuity in satisfying the children's desire to do something, the American exhibits "take the cake." After all, that is the grand secret of toys, and this desire lies at the bottom of much of that vicious determination to get into the middle of things which makes the donors of expensive presents shudder. The World's Tower, made up of innumerable laths and pillars, crowned with flags and stretching half way to the nursery ceiling will be for ever in process of building; the American depôt train running on twelve feet of lines into a real station will know few intervals of rest, and the doll's bedstead which comes to pieces and folds up and makes into a wardrobe will for ever be swaying between its two forms of existence. Santa Claus is a fine figure driving reindeer four-in-hand with a load of presents at his back, and the strong joints of himself, his team and his waggon, show that they can undergo as many changes as the despotic owner shall desire. That beautiful Cathedral, too, is all made up of separate blocks, and, to its shame be it spoken, is quite capable of being transformed at a few moments' notice into a barracks or a theatre of varieties.

It is always difficult to get away from a toy stall, but one word must be said as we turn away of the exquisite sound curve tracings shown by Mr. Joseph Gools, of Nottingham, by means of an instrument of his own invention. It is impossible to reproduce here any of the complex and beautiful markings which he obtains; they are interesting not only from their extreme delicacy and curious developments, but also as illustrating the possible combinations of the elements of harmonic sounds. It may be noted that the sounds to which these twin eliptic tracings correspond in harmony are five octaves below the ordinary range of hearing.

* Movable Picture Books. Dean and Son.
† Fry's Chocolate Nursery Rhymes. Dean and Son.
‡ Old Mother Hubberd of 1793. Dean and Son.

## Figures and Tables

**Figure f1:**
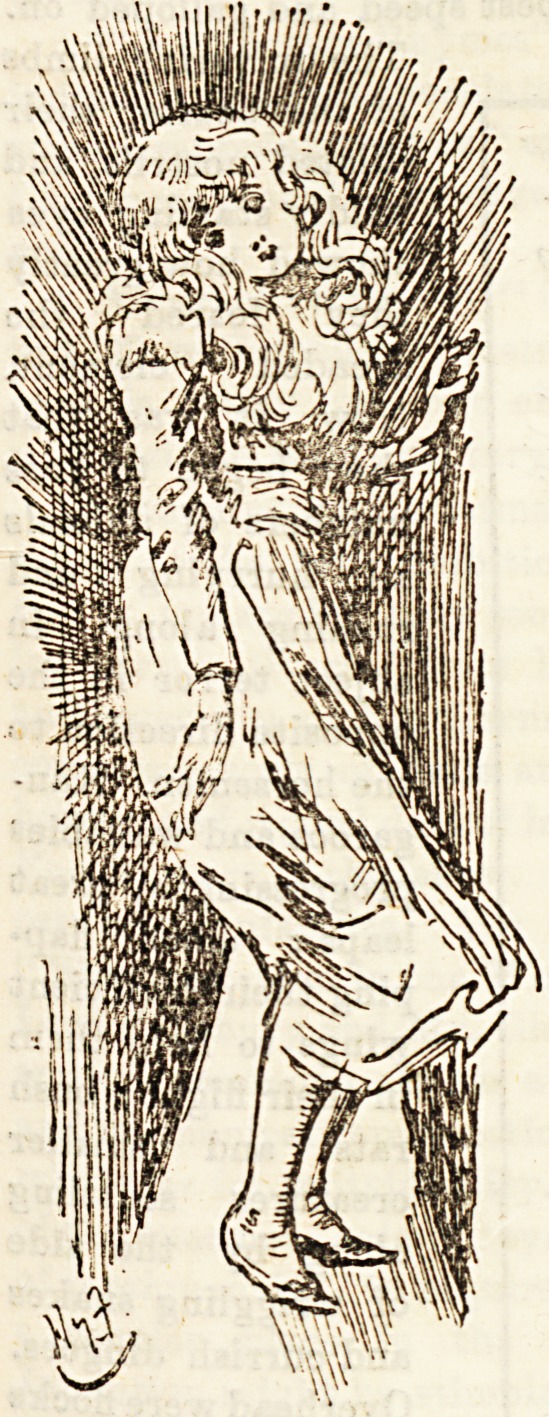


**Figure f2:**
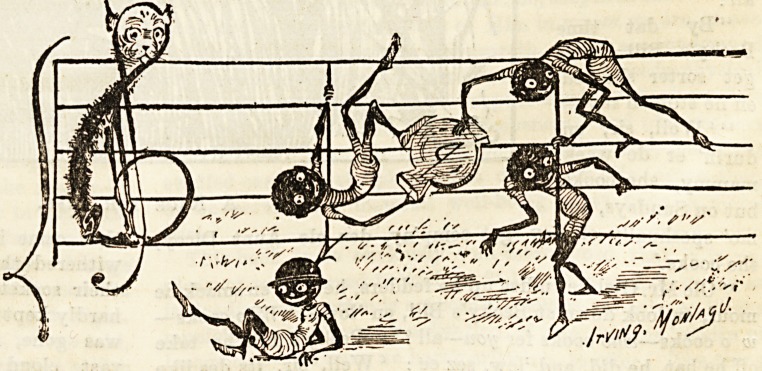


**Figure f3:**